# Gradual loaded exercise of knee extension muscles using an orthosis after wide resection of a femoral sarcoma and quadriceps muscle: a case report

**DOI:** 10.1186/s13256-023-04165-9

**Published:** 2023-10-17

**Authors:** Ippei Kitade, Hisashi Oki, Takumi Sakamoto, Akihiko Matsumine

**Affiliations:** 1 Division of Rehabilitation Medicine, University of Fukui Hospital, 23-3 Shimoaizuki, Matsuoka, Eiheiji-cho, Yoshida-gun, Fukui, 910-1193 Japan; 2 Department of Orthopaedic Surgery, University of Fukui, 23-3 Shimoaizuki, Matsuoka, Eiheiji-cho, Yoshida-gun, Fukui, 910-1193 Japan

**Keywords:** Soft-tissue sarcoma, Quadriceps muscle, Wide resection, Orthosis, Case report

## Abstract

**Background:**

Details of improved gait ability after wide resection of soft tissue sarcomas that necessitate removal of portions of the quadricep muscle have not yet been reported. We describe a patient with improved gait ability following a rehabilitation program after wide resection of a soft tissue sarcoma that included four components of the quadricep muscle.

**Case presentation:**

An 85-year-old Japanese man underwent wide resection of an undifferentiated pleomorphic sarcoma that included portions of the quadriceps femoris muscle. The rectus femoris, vastus medialis, sartorius, and vastus intermedius were separated in the maximally bulging region of the tumour. Three weeks postoperatively, gait exercise was initiated using a rigid knee orthosis with a dual-adjustable lock knee. The contraction loading of the knee extension muscle was controlled by adjusting the hinge motion range of the orthosis as follows: fully extended, fixed knee 0°–30°, and free range. Under this regimen, he could walk independently without a rigid orthosis within 5 weeks postoperatively but could not sit on his heels during daily living activities. At six months, there was no clinical evidence of recurrent tumours or complications.

**Conclusions:**

Postoperative gait ability might be affected by not only the number of resected muscles but also by the function of the separated muscles and the cross-sectional area of the remaining muscle. Gradually loaded exercise of the knee extension muscles using an orthosis could result in an improved gait motion for patients who undergo wide resection of a sarcoma that includes four components of the quadriceps femoris.

## Background

Surgical options for femoral soft tissue sarcomas (STSs) include amputation and limb salvage. As limb salvage surgery is associated with an improved quality of life [1, 2], and has similar local recurrence and cumulative survival rates [3, 4] compared to amputation, it has become a preferred option for patients with malignant musculoskeletal tumours. There are also reports that the surgeon’s first and foremost goal should be the wide resection of sarcomas [5, 6]. DeVissar *et al.* [7] described gait adaptations of limb salvage procedure and expectedly reported that the restoration of gait after limb salvage is impressive, but not complete. Although several studies have described the relationship between gait ability and rehabilitation programs [8–10], studies highlighting an improvement in gait ability following rehabilitation after tumour-wide resection are limited. Shehadeh *et al.* [8] described outcomes of standardised site-specific rehabilitation after limb salvage surgery for sarcomas. Additionally, a few rehabilitation procedures for patients after wide resection have previously been described. However, all the patients in such rehabilitation procedures, underwent wide resection with artificial bone joint [8–14]. Furthermore, removal of the quadriceps also disrupts the knee extension mechanism, resulting in knee instability and impairment of activities of daily living, including walking [13, 15, 16]. However, we found no detailed reports of acute rehabilitation procedures after wide resection, without an artificial bone joint.

We describe the case of a patient with STS who underwent wide resection that included four components of the quadriceps muscle. Postoperatively, he underwent a rehabilitation regimen using rigid knee orthosis and achieved a high level of gait function. Here, we discuss the key points of the acute rehabilitation procedure that resulted in positive outcomes for this patient.

## Case presentation

**Fig. 1:**
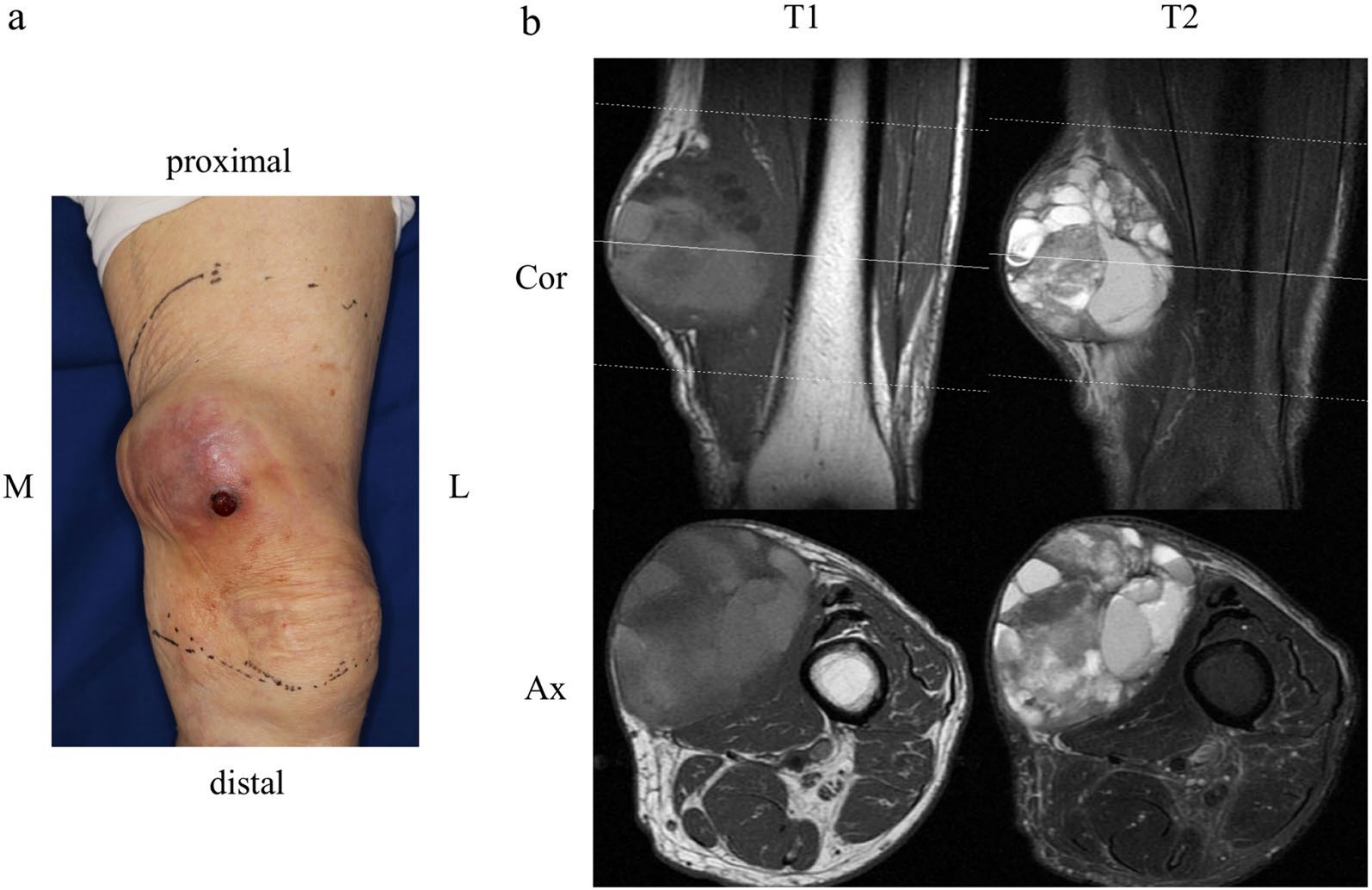
Preoperative physical condition **a** and magnetic resonance imaging scans **b** of the femur. **a** Left medial femur with a tumour mass. **b** Soft tissue mass with internal heterogeneity, cystic degeneration, and fluid–fluid level. *M* medial, *L* lateral, *T1* T1-weighted images, *T2* T2-weighted images, *Cor* coronal, *Ax* axial

**Fig. 2:**
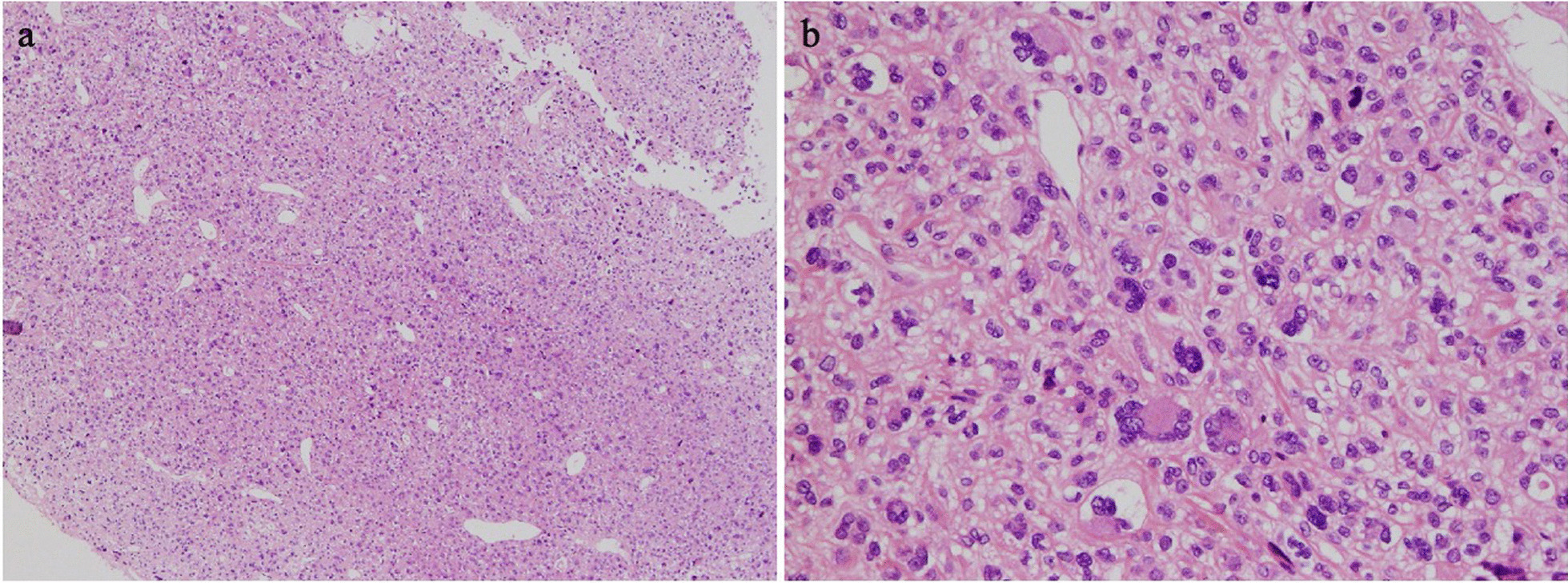
Histological findings showed that the tumour grew mainly in the subcutaneous adipose tissue and was covered with a fibrous capsule, but infiltration outside the capsule was also partially observed (**a** H&E × 40). Tumour cells had various prominent nuclear atypia such as spindle-shaped and large round, and some had mononuclear, polynuclear, and malformed nuclei (**b** H&E × 200). Immunohistochemical studies revealed, positive staining with vimetin. Staining with Cytokeratin AE1/AE3, EMA, desmin, α-SMA, S-100, CD34 and CD99 were negative

An 85-year-old Japanese man had a 10-year history of a tumour mass in the left femur that had gradually enlarged over the past 2 years. He could walk independently without any support (10 m-walk test 14.6 s) and could perform daily living activities normally (manual muscle test of the bilateral leg 5/5, functional independence measure 126/126 points). His cognitive function was normal (Mini-Mental State Examination score, 30/30 points). T2-weighted magnetic resonance imaging showed a 90 × 75 × 65 mm soft tissue mass of inhomogeneous intensity that contained an area of cystic degeneration with a fluid–fluid level at the medial side of the tumour (Fig. 1a, b). Needle biopsy revealed an undifferentiated pleomorphic sarcoma (Fig. 2).

**Fig. 3:**
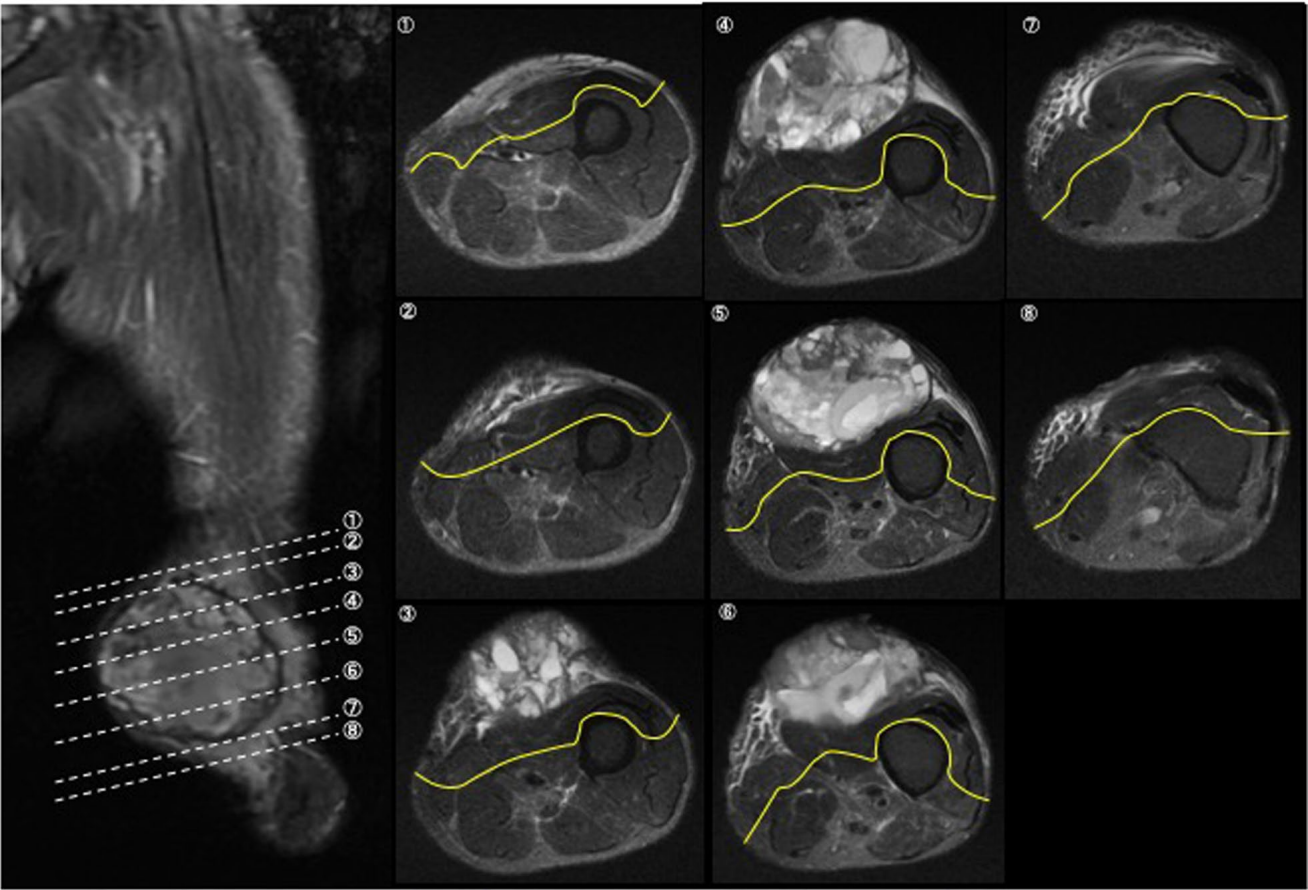
Procedure for wide resection of the femur. Solid (yellow) lines are resection lines on T2-weighted images. En bloc wide resection of the sarcoma included the quadriceps femoris and skin. Tumours were severed 20 mm proximally, 20 mm distally, and 20 mm dorsally and included the reactive layer. The rectus femoris, vastus medialis, sartorius, and vastus intermedius were separated at the tumour’s maximally bulging region. A portion of the vastus lateralis was excised. 1–8 (lower left) shows the levels at which the resections were performed

Therefore, the patient underwent wide resection of the sarcoma that included part of the quadriceps femoris and the skin (Fig. 3). Figure 3 depicts the procedural details for wide resection of the femur and resection lines. The tumour was severed 20 mm proximally, 20 mm distally, and 20 mm dorsally, and included the reactive layer. The rectus femoris, vastus medialis, sartorius, and vastus intermedius were separated in the maximally bulging region of the tumour. In addition, a portion of the vastus lateralis was excised, with the remainder retained to maintain continuity in the long-axis direction. The skin defect was reconstructed using a free-skin graft. The total blood loss during the surgery was 192 g. The patient did not undergo adjuvant treatment (for example, irradiation therapy and chemotherapy) before or after surgery.

**Fig. 4:**
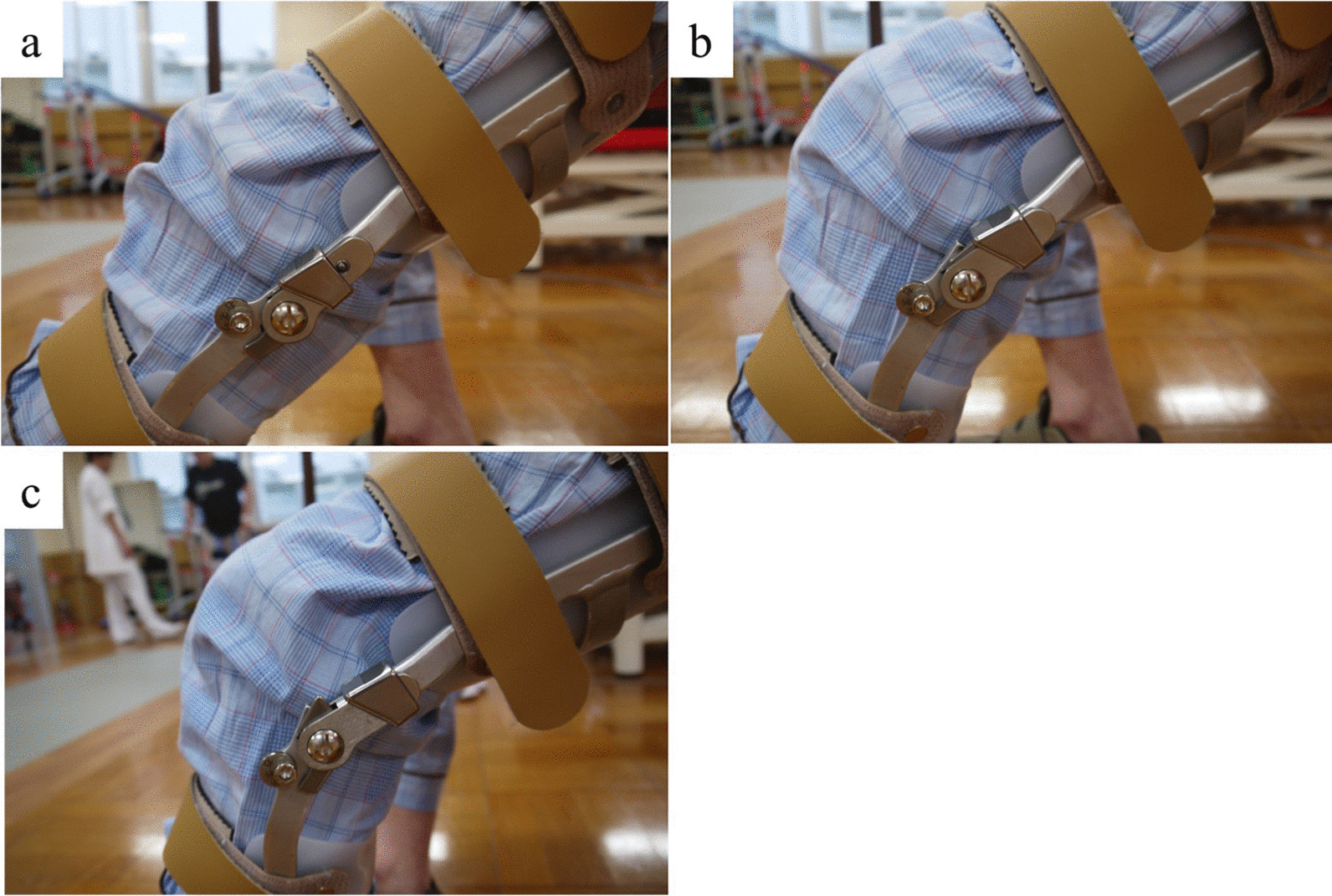
Rigid knee orthosis with dual-adjustable lock knee. **a** Fully extended fixed knee. **b** Range 0°–30°, which allowed 0°–30° of knee range of motion (ROM). **c** Free range (no limitation of knee ROM)

On postoperative day 5, he was started on a rehabilitation (physical therapy) program that included range of motion (ROM) exercises, muscle strengthening, and single-leg standing balance exercises of the bilateral leg. Gait exercises were started three weeks postoperatively using a rigid knee orthosis with a dual-adjustable lock knee (Keiai Orthopedic Appliance Co., Ltd., Saitama, Japan). The contraction loading of the knee extension muscle was controlled by adjusting the hinge ROM on the orthosis as follows: fully extended, fixed-knee 0°–30° (which allowed 0°–30° of knee ROM), and free range (no limitation of knee ROM) (Fig. 4). Gudas [13] reported that early gait training and weight bearing are accepted approaches for patients following a wide resection. However, early aggressive and passive range-of-motion exercises are unwise, and isometric exercises are beneficial within the active-assisted range. Therefore, we investigated an orthosis that could control the contraction load of the knee extensor muscles by adjusting the hinge ROM of the orthosis. At 3 weeks, he also started gait exercise with a cane and orthosis under the fully extended knee condition, followed by gait exercise without a cane and with the orthosis at 0°–30°. Four weeks postoperatively, he was allowed to walk under the free-range condition of the knee joint. Following this progression, at 5 weeks, he could walk without a knee orthosis and had no knee buckling (10 m-walk test 16.6 s).

Although the Musculoskeletal Tumor Society (MSTS) score [17] at 3 weeks postoperatively was 23.3%, it improved to 76.7% by the 5^th^ week (Table 1). However, the “function” score was only 1 point at the 5-week postoperative follow-up because he had to change from a normal squatting position to a sitting position during toileting self-care, as he could not achieve a deep flexion angle at the knee joint. The Functional Independence Measure score at 1 week after surgery was 101 points but improved to 122 points at 5 weeks (Table 1).

**Table 1:** Clinical parameters before and after wide resection

	Before surgery	After surgery			
		1 week	3 weeks	4 weeks	5 weeks
MSTS score (points)					
Total (points (%))	29 (96.7)	–	7 (23.3)	14 (46.7)	23 (76.7)
Pain	5	–	3	4	5
Function	5	–	1	1	1
Emotional	4	–	1	2	3
Supports	5	–	1	3	4
Walking	5	–	0	1	5
Gait	5	–	1	3	5
FIM score (points)					
Total	126	101	–	–	122
Motor subtotal rating	91	66	–	–	87
Self-Care					
Eating	7	7	–	–	7
Grooming	7	7	–	–	7
Bathing	7	1	–	–	5
Dressing-upper body	7	7	–	–	7
Dressing-lower body	7	5	–	–	7
Toileting	7	5	–	–	7
Sphincter control					
Bladder management	7	7	–	–	7
Bowel management	7	7	–	–	7
Transfers					
Bed, Chair, Wheelchair	7	5	–	–	7
Toilet	7	6	–	–	7
Tub, Shower	7	1	–	–	6
Locomotion					
Walk/Wheelchair	7	7	–	–	7
Stairs	7	1	–	–	6
Cognitive subtotal score	35	35	–	–	35
Communication					
Comprehension	7	7	–	–	7
Expression	7	7	–	–	7
Social cognition					
Social interaction	7	7	–	–	7
Problem solving	7	7	–	–	7
Memory	7	7	–	–	7

*MSTS* Musculoskeletal Tumor Society, *FIM* Functional Independence Measure

**Fig. 5:**
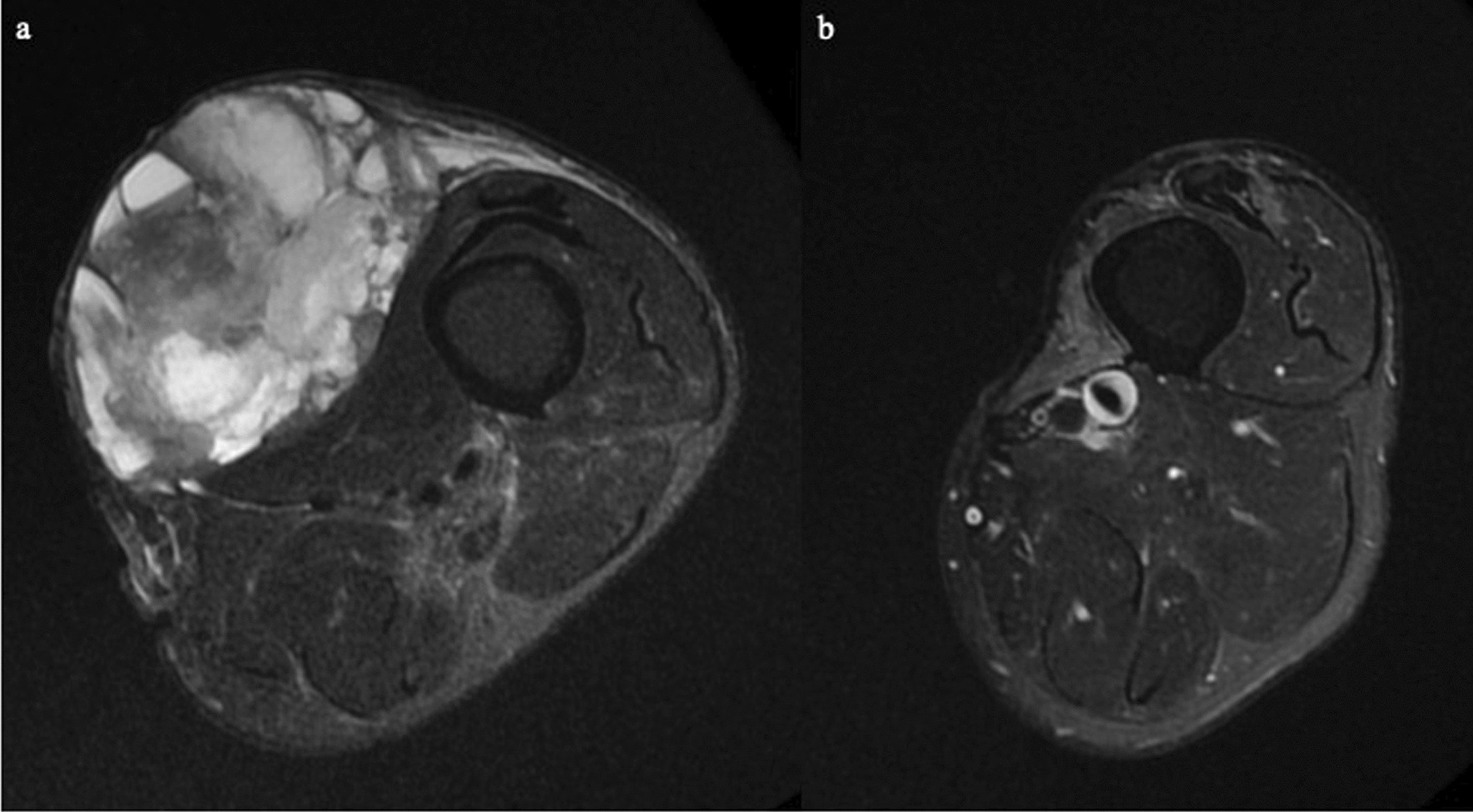
Cross-sectional areas of the quadriceps muscle in the tumour’s maximally bulging region. **a** Before surgery it measured 2434.4 mm^2^ (rectus femoris, vastus medialis, vastus intermedius, and vastus lateralis). **b** Six months after surgery it was 921.7 mm^2^ (portion of the vastus lateralis is visible). This 6-month postoperative cross-sectional area of the quadriceps muscle (at the level of longest diameter of the tumour) had decreased to 37.9% of that before surgery

Six months after the wide resection, he had no pain in the left leg, could accomplish daily living activities independently, and walked independently without orthosis or a cane. At the 6-month follow-up evaluation, the knee flexion angle was 150° and the MSTS score also substantially improved. The cross-sectional area of the quadriceps muscle (the portion of the vastus lateralis) at 6 months postoperatively decreased to 37.9% of the original area before surgery [2434.4 mm^2^ (before) vs 921.7 mm^2^ (after)] and was at the level of the longest diameter of the tumour (Fig. 5). There was no reduction in the daily living activities until 18-months after surgery. However, multiple lung metastases were observed at 21-months after surgery, and we were unable to follow up the symptoms from that point.

## Discussion

Wide resection is the standard surgical procedure for patients with malignant musculoskeletal tumours. However, wide resection of a large tumour sometimes causes severe muscle weakness and gait disturbance. Yamaguchi *et al.* [18] showed that low MSTS scores were correlated with large muscular defects, which especially affected function, walking ability, and gait appearance scores. Benedetti *et al.* [11] reported that patients who underwent resection of the vastus lateralis and vastus intermedius had better gait performance and a more physiological knee-loading pattern than those with vastus medialis resection. Although our patient underwent wide resection of the sarcoma, including four components of the quadriceps femoris (only part of the vastus lateralis was retained to maintain continuity in the long-axis direction), he showed acceptable limb function 6 weeks after the surgery. Additionally, although the cross-sectional area of the quadriceps femoris (part of the vastus lateralis) was reduced to approximately 38% of the preoperative level at 6-month after surgery, acceptable limb function was maintained at 18 months postoperatively. The proximal portion of the resected quadriceps muscle could adhere to the remaining muscle [15] and thus work as an extension of the muscle.

Henshaw *et al.* [12] reported that, despite widespread agreement on the goals of rehabilitation following limb salvage, the actual rehabilitation guidelines that patients should follow remain scarce and unreported. Gudas [13] reported that early gait training and weight bearing are accepted approaches for patients after wide resection. Early aggressive passive range-of-motion exercises are unwise, and isometric exercises are beneficial within the active-assisted range. However, rehabilitation techniques for patients after wide resection, remains conjectural and largely untested [13]. Therefore, we investigated an orthosis that could control the contraction load of the knee extensor muscles by adjusting the hinge ROM of the orthosis. There were few previous studies reporting rehabilitation procedures [8–14], however, all patients in those studies underwent wide resection using artificial bone joints. We were unable to find any study detailing acute rehabilitation procedures after wide resection without an artificial bone joint. Patients who undergo wide resection with artificial bone joints are prepared for aggressive postsurgical rehabilitation to prevent arthrofibrosis [19]. Therefore, it is difficult to compare the programs and outcomes of our study with that of previous studies given the differences between acute phase rehabilitation programs, with and without artificial bone joints. Hence, our report is the first to provide a detailed acute rehabilitation regimen for improving gait motion in patients who undergo wide resection of an STS that includes four components of the quadriceps femoris without an artificial bone joint. Scientific analysis of postoperative limb function is difficult because there are several variables that could affect postoperative limb function, such as the number, length, and combination of the resected muscles; residual motor nerve with or without bone resection; reconstruction procedure of the bone and soft tissue defect; and patient age. Hence, we analysed the details of postoperative gait ability and limb function in a patient who underwent wide resection of the STS that necessitated removal of the distal portion of the four components of the quadriceps femoris.

We propose a rehabilitation that includes a rigid knee orthosis with a dual-adjustable dial lock knee for patients who undergo wide resection of the STS along with the distal portion of the quadriceps femoris. Reilly and Martens [20] reported that the output of the quadriceps muscle increases as the knee joint flexion angle increases under the weight-bearing conditions of the leg. Our patient would have therefore been predicted to fall down or show buckling of the knee resulting from decreased muscle strength due to his oldest-old age category and the wide resection procedure [13]. On the contrary, the patient achieved relatively stable gait ability and acceptable ability to perform daily living activities because his rehabilitation regulated the load on the quadriceps muscle by the use of an orthosis with adjustable brakes and angles.

## Conclusions

We suggest that the gradual load exercise of the knee extension muscles using the orthosis might result in improved gait motion for patients who undergo wide resection of STS, including the removal of four components of the quadriceps femoris. As a limitation, our presentation is a case report. Hence, a study with an increased number of patients, assessing further quantitative parameters, such as three-dimensional gait analysis, is needed. In this case, the orthosis was delayed because of the large STS. If the orthosis was finished early, he might have started gait exercises earlier than three weeks after surgery.

## Data Availability

Not applicable.
